# Annual acknowledgement of manuscript reviewers

**DOI:** 10.1186/1746-4269-10-17

**Published:** 2014-02-08

**Authors:** Andrea Pieroni

**Affiliations:** 1 University of Gastronomic Sciences, Piazza Vittorio Emanuele 9, 12060 Bra/Pollenzo, Cuneo, Italy

## Abstract

**Contributing reviewers:**

The editors of *Journal of Ethnobiology and Ethnomedicine* would like to thank all our reviewers who have contributed to the journal in Volume 9 (2013).

## 

Arshad Abbasi

Pakistan

Raziq Abdul

Pakistan

Talal Aburjai

Jordan

Michael Adams

Switzerland

Getachew Addis

Ethiopia

Ulysses Paulinho de Albuquerque

Brazil

Romulo Alves

Brazil

Tinde van Andel

Netherlands

Myrdene Anderson

United States of America

Tamar Bajgielman

Brazil

Flavio Barros

Brazil

Alpina Begossi

Brazil

Shandesh Bhattarai

Nepal

Hugo de Boer

Sweden

Alessandro Boesi

Italy

Piero Bruschi

Italy

Rainer W Bussmann

United States of America

Anja Byg

United Kingdom

Sophie Caillon

France

Ana Maria Carvalho

Portugal

David Casagrande

United States of America

Alejandro Casas

Mexico

Hector Castaneda

El Salvador

Anthony Cavender

United States of America

Giacomo Certini

Italy

Melissa Ceuterick

United Kingdom

Ahmad Cheikhyoussef

Namibia

Mariana Clauzet

Brazil

Claire Couly

French Guiana

Gisella S Cruz-Garcia

Colombia

Maria de los Angeles La Torre Cuadros

Peru

Anthony Davis

Canada

Shivcharn S Dhillion

Norway

Yunus Dogan

Turkey

Pablo Dominguez

Spain

Roy Ellen

United Kingdom

Kakudidi Esezah

Uganda

Eduardo Estrada

Mexico

Veerle Van den Eynden

United Kingdom

Michele Filippo Fontefrancesco

Italy

Adediwura Fred-Jaiyesimi

Nigeria

Natalie Friend-du Preez

United Kingdom

Takeshi Fujimoto

Japan

Rupo T Gahukar

India

Orou Gaoue

United States of America

Gisella Cruz Garcia

United States of America

Roberto Garibay-Orijel

Mexico

Sjaak van der Geest

Netherlands

Abdolbaset Ghorbani

Iran

Mirutse Giday

Ethiopia

Peter Giovannini

United Kingdom

Erik Gómez-Baggethun

Spain

José Antonio González

Spain

Maria Reyes González-Tejero

Spain

Andrew Gosler

United Kingdom

Maximilien Gueze

Spain

Karl Hammer

Germany

Natalia Hanazaki

Brazil

Norma Hilgert

Argentina

Anita Jain

India

Hannah Jennings

United Kingdom

Leslie Main Johnson

Canada

Chandra Prakash Kala

India

Yongxiang Kang

China

Jasper Kenter

United Kingdom

Hafiz Azhar Ali Khan

Pakistan

Shujaul Mulk Khan

Pakistan

Khasbagan

China

Akhilesh Kumar

India

Ana Haydee Ladio

Argentina

Frank Lake

United States of America

Cheryl Lans

Canada

Marco Leonti

Italy

Nicolas Lescureux

Norway

Chunlin Long

China

Lukasz Luczaj

Poland

Ermias Lulekal

Ethiopia

Ana Luz

Spain

Manuel J Macia

Spain

Alfred Maroyi

South Africa

Gary Martin

Morocco

Viviane Martins

Brazil

Sarah-Lan Mathez-Stiefel

Switzerland

Firew Mekbib

Ethiopia

Matthias F Melzig

Germany

Rachel Meyer

United States of America

Victor Benno Meyer-Rochow

Germany

Adriana Montoya

Mexico

Ángel Moreno-Fuentes

Mexico

Mainen Moshi

Tanzania

Nemer Narchi

Mexico

Oraib Nawash

Jordan

Eraldo Costa Neto

Brazil

Steven Newmaster

Canada

Sanjoy Ningthoujam

India

Justin Nolan

United States of America

Olawole Obembe

Nigeria

Manuel Pardo-de-Santayana

Spain

Wendy Pearson

Canada

James Penn

United States of America

Charles Peters

United States of America

Lisa Aston Philander

United States of America

Heidemarie Pirker

Austria

Martin Potgieter

South Africa

Mariève Pouliot

Denmark

Lisa Leimar Price

Netherlands

Marsha Quinlan

United States of America

Subramanyam Ragupathy

Canada

Mohammed Rahmatullah

Bangladesh

Julieta Ramos-Elorduy

Mexico

Angela Ratsch

Australia

Victoria Reyes-Garcia

Spain

Nanci Ross

United States of America

Norbert Ross

United States of America

Ian Rotherham

United Kingdom

Jan Salick

United States of America

Haris Saslis-Lagoudakis

Australia

Azusa Sato

United Kingdom

Valentina Savo

Canada

Sebua Semenya

South Africa

Charlie Shackleton

South Africa

Pei Shengji

China

Glenn H Shepard Jr.

Brazil

Krishna Shrestha

Nepal

Stephanie Shwiff

United States of America

Maria Adele Signorini

Italy

Paul Sillitoe

United Kingdom

Renato Silvano

Brazil

Vania Smith-Oka

United States of America

Renata Soukand

Estonia

John Richard Stepp

United States of America

Ingvar Svanberg

Sweden

John RS Tabuti

Uganda

Tilahun Teklehaymanot

Ethiopia

Evert Thomas

Belgium

Alain Touwaide

United States of America

Chusie Trisonthi

Thailand

Nancy Turner

Canada

Joan Vallès

Spain

Ina Vandebroek

United States of America

Robert Voeks

United States of America

Christian R Vogl

Austria

Gabriele Volpato

Netherlands

Wycliffe Wanzala

Kenya

Helene de Wet

South Africa

Merlin Willcox

United Kingdom

Ben-Erik Van Wyk

South Africa

Erdem Yesilada

Turkey

Marius Yetein

Benin

Delenasaw Yewhalaw

Ethiopia

Haile Yineger

Ethiopia

Hadar Zaman

United Kingdom

Rebecca Zarger

United States of America

Stanford Zent

Venezuela

